# Current approach to genetic testing and genetic evaluation referrals for adults with congenital heart disease

**DOI:** 10.3389/fgene.2024.1398887

**Published:** 2024-05-13

**Authors:** Laura B. Oehlman, Alexander R. Opotowsky, Kathryn N. Weaver, Nicole M. Brown, Cara L. Barnett, Erin M. Miller, Hua He, Amy R. Shikany

**Affiliations:** ^1^ Division of Human Genetics, Cincinnati Children’s Hospital Medical Center, Cincinnati, OH, United States; ^2^ Department of Pediatrics, University of Cincinnati College of Medicine, Cincinnati, OH, United States; ^3^ Department of Medical and Molecular Genetics, Indiana University School of Medicine, Indianapolis, IN, United States; ^4^ Heart Institute, Cincinnati Children’s Hospital Medical Center, Cincinnati, OH, United States; ^5^ Cincinnati Children’s Hospital Medical Center, Cincinnati, OH, United States

**Keywords:** cardiology, genetic testing, inclusion of genetic services, ACHD, clinical practice

## Abstract

**Background:**

Congenital heart disease (CHD) is the most common congenital anomaly. Up to 33% have an identifiable genetic etiology. Improved medical and surgical management of CHD has translated into longer life expectancy and a rapidly growing population of adults living with CHD. The adult CHD (ACHD) population did not have access during childhood to the genetic technologies available today and therefore have not had a robust genetic evaluation that is currently recommended for infants with CHD. Given this potential benefit; the aims of this study were to determine how ACHD cardiologists offer genetics services to patients and identify the indications that influence decision-making for genetics care.

**Methods:**

We performed a descriptive cross-sectional study of ACHD cardiologists. A study-developed questionnaire was distributed via emailed REDCap link. The recruitment email was sent to 104 potential respondents. The survey was open from 06/2022 to 01/2023.

**Results:**

Thirty-five cardiologists participated in the study (response rate of 34%). Most cardiologists identified as white (77%) and male (66%). Cardiologists were more likely to refer patients to genetics (91%) than to order testing themselves (57%). Of the testing ordered, chromosomal testing (55%) was ordered more than gene sequencing (14%). Most cardiologists would refer a patient with a conotruncal lesion (interrupted aortic arch) over other indications for a genetics evaluation. There were more reported barriers to ordering genetic testing (66%) compared to referring to genetics for a genetics evaluation (23%). Cardiologists were more confident recognizing features suggestive of a genetic syndrome than ordering the correct test (*p* = 0.001). Regarding associations between clinical factors and current practices, more years in practice trended towards less referrals and testing. Evaluating a greater number of patients (*p* = 0.11) and greater confidence recognizing syndromic features (*p* = 0.12) and ordering the correct test (*p* = 0.09) were all associated with ordering more testing.

**Conclusion:**

Testing for microdeletion syndromes is being offered and completed in the ACHD population, however testing for single-gene disorders associated with CHD is being under-utilized. Developing guidelines for genetic testing in adults with CHD could increase access to genetic services, impact medical management, reduce uncertainty regarding prognosis, and inform recurrence risk estimates.

## 1 Introduction

Congenital heart disease (CHD) has a birth incidence of 0.8%–1.0% making it the most common major congenital anomaly seen in newborns (Bracher et al., 2017; Ito et al., 2017).

Most heart defects are isolated and have an unknown etiology, but up to 33% of patients with CHD have an identifiable genetic cause (De Backer et al., 2020). Genetic etiologies for CHD can be divided into chromosomal anomalies and single gene variants. Chromosomal associated CHD includes trisomy 21 (Down syndrome), monosomy X (Turner Syndrome), and microdeletions at 22q11.2 (DiGeorge, velocardiofacial syndrome), 7q11.23 (Williams Syndrome) and 1p36. Single-gene variants can cause syndromic CHD (e.g., Noonan syndrome, Holt-Oram syndrome, Alagille syndrome) as well as isolated genetic CHD (e.g., variants in NOTCH1 and FLT4) (Pierpont et al., 2007; Nees and Chung, 2020). Patients with CHD benefit from evaluation for chromosomal abnormalities, single gene variations, congenital exposures, and multi-system associations such as VACTERL (a disorder including vertebral defects, anal atresia, cardiovascular anomalies, tracheoesophageal fistula, esophageal atresia, renal anomalies, and limb defects) (Bracher et al., 2017).

Historically, infants with syndromic CHD were diagnosed based on characteristic facial features and symptoms resembling cohorts of other patients with genetic syndromes (Rimoin and Hirschhorn, 2004). More recently, diagnostic practices have shifted to identifying chromosomal and gene variations through karyotype, fluorescent *in situ* hybridization (FISH), chromosomal microarray (CMA), and next-generation sequencing (NGS) techniques (genetic panels, whole exome sequencing (WES), and whole genome sequencing (WGS)) (Pierpont et al., 2007). The benefits and limitations of genetic testing for patients with CHD affect how individuals and families are counseled and can be critical to the medical diagnosis and management of CHD (De Backer et al., 2020).

Confirming a genetic cause for a heart defect can reduce uncertainty and worry about a prognosis, inform recurrence risk estimates, and can impact medical management (Bernier and Spaetgens, 2006). For example, genetic testing can lead to the identification of individuals who are at risk for comorbidities such as heart failure, arrhythmias, and neurodevelopmental disorders (NDD). Understanding the cause of a heart defect can also guide cardiac screening (electrocardiograms, echocardiograms, etc.), screening of other organ systems, involvement of necessary specialists (vascular surgery, endocrinology, etc.) and timely interventions, and in the future, targeted curative therapies (De Backer et al., 2020). When considering preconception decision-making, meeting with a genetics provider can help families understand etiology and family risks and may influence when and how individuals with a history of CHD have children (van Engelen et al., 2013).

Medical advances have led to improved diagnosis and management of adults with congenital heart disease (ACHD). Two-dimensional echocardiograms first became available in the 1970s and greatly improved the diagnosis of CHD (Kiess, 2016; Lapum et al., 2019). Improving medical and surgical management of CHD has translated into longer life expectancy; with approximately 90% of patients with CHD born after 1990 having survived to adulthood. There is now a rapidly growing population of adults living with CHD (Mazor Drey and Marelli, 2015). Many of these adults did not have access during childhood to genetic technologies available today and therefore have not had a robust genetic evaluation. We propose that this population would benefit from the same genetic testing and counseling that is currently considered for infants with CHD (Parrott and Ware, 2012; Stout et al., 2019).

Given the potential benefit of genetic evaluation of adults with CHD, we sought to determine how Adult Congenital Heart Disease (ACHD) cardiologists currently approach offering genetic testing and genetic evaluations to patients with ACHD. We also sought to evaluate the indications and practice structures that influence decision-making by ACHD cardiologists when it comes to the provision of genetics care. We hypothesized that adults with CHD are not receiving indicated genetics services and that there is a need for concise and clear guidelines regarding genetic evaluation and testing in the ACHD population.

## 2 Materials and methods

### 2.1 Study population

This descriptive cross-sectional study surveyed cardiologists who self-reported that they provide care to adults with congenital heart disease (CHD). Approval from the Institutional Review Board at Cincinnati Children’s Hospital Medical Center (CCHMC) was obtained (2022-0324). The survey email list was obtained from the Adult Congenital Heart Association. Cardiologists that self-reported being a currently practicing board-certified cardiologist were eligible to participate in the study. Physicians in other specialties and non-patient-facing cardiologists were excluded from the study.

### 2.2 Survey distribution

An invitation for participation in the survey was emailed to ACHD cardiologists using REDCap and included a personal link and URL to the questionnaire. Initially, the survey was emailed to the first 20 emails alphabetically to ensure cardiologists were able to access and answer the questionnaire. Three completed surveys were submitted from the first group before the email was sent to the remainder of the email list. The recruitment email was sent to 113 total ACHD cardiologists. Eight emails could not be delivered and we received one request asking to be removed from the email list. This left 104 potential respondents. The survey was open from June 2022 to January 2023. Three reminder emails were sent to individuals on the email list who had not completed the survey.

### 2.3 Questionnaire development

A questionnaire was compiled for the purpose of the study and is available in the supplementary materials. The questionnaire is not validated. The questions were based on an article by Boynton and Greenhalgh (2004) on selecting, designing, and developing a questionnaire as well as questions used by other specialties for similar studies (Prochniak et al., 2012). The questions were organized into 7 categories in the following order: screening questions, demographics, access to genetics professionals, confidence with genetic knowledge and testing, using genetic testing, making genetics referrals, and perspectives on use of genetics in practice. The questionnaire was administered through REDCap hosted at CCHMC and skip-logic was utilized to avoid irrelevance and redundancy of questions (Harris et al., 2009 and 2019). The questionnaire was pre-tested by two ACHD cardiologists at CCHMC for question content and clarity.

### 2.4 Data analysis

The data from participation in the questionnaire was exported from REDCap. Descriptive statistics were used to characterize the study population. Frequency (percentage) was reported for all categorical variables. Median and inter-quartile range (IQR) was reported for continuous variable (knowledge and confidence scores). Fisher’s exact tests were used to examine the associations between testing/referral practices and clinical factors (cardiologist’s level of training and clinical experience). Knowledge score was calculated using three case-based knowledge questions. Cardiologists scored one point per question they answered correctly. The third question asked to select more than one patient and each option was assigned a quarter of a point. The knowledge score ranged from 0 to 3. For the confidence questions, numerical values were assigned to Likert answer scores (“not confident at all” = 1; “slightly confident” = 2; “Neutral” = 3; “fairly confident” = 4; “completely confident” = 5). The confidence score ranged from 1 to 5. Wilcoxon rank-sum tests were used to test association between knowledge/confidence in genetic testing and testing/referral practices. Given the exploratory nature of this study and our limited sample size, a *p*-value threshold of *p* < 0.2 was applied for significance. All the analyses were performed in R software, version 4.2.0 (GNU Project, Free Software Foundation, https://www.r-project.org).

## 3 Results

Of the 104 ACHD cardiologists who received an invitation to participate in the study, 35 cardiologists participated and met the inclusion criteria of the study (response rate of 34%). Two cardiologists did not finish the survey but their partial responses were included in data analysis.

### 3.1 Demographics

Most cardiologists identified as white (77%) and male (66%). All cardiologists had a Doctor of Medicine (M.D.) degree and four cardiologists (11%) had more than one advanced degree. Most cardiologists completed a fellowship in ACHD (89%), currently work at an academic medical center (89%), and practice in the United States (66%). Table 1 contains a complete list of cardiologist demographics.

**TABLE 1:** Cardiologist demographics and training.

	N (n = 35)	%
Gender		
Male	23	66
Female	11	31
Self-identify—*free text*	0	0
No response	1	3
Ethnicity—*Could select more than one option*		
White	27	77
Black or African American	0	0
Hispanic or Latino	3	9
Asian	1	3
Other	3	9
No response	2	6
Degree—*Could select more than one option*		
Medical Doctor (MD)	35	100
Doctor of Osteopathy (DO)	0	0
Master of Public Health (MPH)	1	3
Master of Medical Science (MMSc/MMS)	2	6
Other—*free text*		
Master of Science in Clinical Investigation	1	3
ACHD Fellowship		
Yes	31	89
No	4	11
Years since training		
0–5 years	9	26
6–10 years	10	29
11–15 years	9	26
16–20 years	2	6
>20 years	4	11
No response	1	3
Work at an academic medical center		
Yes	31	89
No	2	6
No response	2	6
Country where workplace is located		
United States	23	66
Puerto Rico	1	3
Canada	3	9
Other (One each from Australia, Chile, Saudi Arabia, South Africa, Switzerland)	5	14
No response	3	9

### 3.2 Utilization of genetics professionals and testing services

#### 3.2.1 Clinic structure

Regarding patient load and utilization of genetics services, most (74%) cardiologists indicated that they are involved in more than 400 appointments for ACHD per year. A subset of cardiologists (60%) also indicated that they are involved with pediatric CHD appointments. Of the thirty-one (89%) respondents that reported having a genetics professional at their institution, only ten (32%) indicated that genetics providers were embedded in their ACHD clinic. Cardiologists reported having less barriers to referring to genetics (77%) than to ordering genetic testing (34%). Of the reported barriers to referring, two were selected by more than one cardiologist: difficulties with the logistics of referring patients and connecting them with a genetics professional and long wait times for a genetic evaluation. The greatest barriers to ordering genetic testing according to cardiologists included no changes to management based on testing (29%), not knowing what test to order (17%), high cost to patients for genetic testing (11%), and not being able to contact a genetics professional (11%). For a complete description of reported clinic structures refer to Table 2.

**TABLE 2:** Clinic structure and barriers.

	N (n = 35)	%
How many ACHD appointments are you involved in per year?		
>400	26	74
301–400	5	14
201–300	1	3
101–200	2	6
1–100	1	3
Do you also evaluate pediatric CHD patients?		
Yes	14	40
No	21	60
How many pediatric CHD appointments are you involved in per year?	(n = 14)	
>400	2	14
301–400	0	0
201–300	2	14
101–200	4	29
1–100	6	43
What is the availability of genetics providers at your primary institution?		
At the same institution	31	89
At a different local institution	3	9
No genetics providers available	0	0
Other—*free text*		
Pediatric genetics cannot evaluate adults, only one adult geneticist	1	3
Are there geneticists or genetic counselors embedded in your clinic?	(n = 31)	
Yes	10	32
No	21	68
How are genetics appointments conducted?		
In person only	5	14
Telehealth only	0	0
Both in person and telehealth	24	69
I do not know	5	14
No response	1	3
Barriers to referring patients to genetics providers		
No barriers	27	77
Barriers—*Could select more than one option*	5	14
There is not a geneticist or genetic counselor in my clinic	1	3
Meeting with genetics would be logistically difficult for patients	2	6
Other—*free text*		
Very long wait to see a genetics provider	2	6
Scheduling is logistically difficult	2	6
Limited availability of genetics professionals	1	3
No response	3	9
Barriers to ordering testing for patients with ACHD		
No barriers	12	34
Barriers—*Could select more than one option*	22	63
I could easily refer to genetics professionals to order testing for me	11	31
I did not know how to order genetic testing	3	9
I did not know the best genetic test to order	6	17
I did not feel confident interpreting genetic testing	3	9
I was not able to contact a geneticist or genetic counselor	4	11
My patients were not interested in genetic testing	1	3
I was concerned about the patient’s risk of genetic discrimination	2	6
Ordering genetic testing would not have changed management	10	29
Other—*free text*		
Concerns about the cost of genetic testing	4	11
Would not change management for post-menopausal patients	1	3
No response	1	3

#### 3.2.2 Reported practices in the last year

**FIGURE 1:**
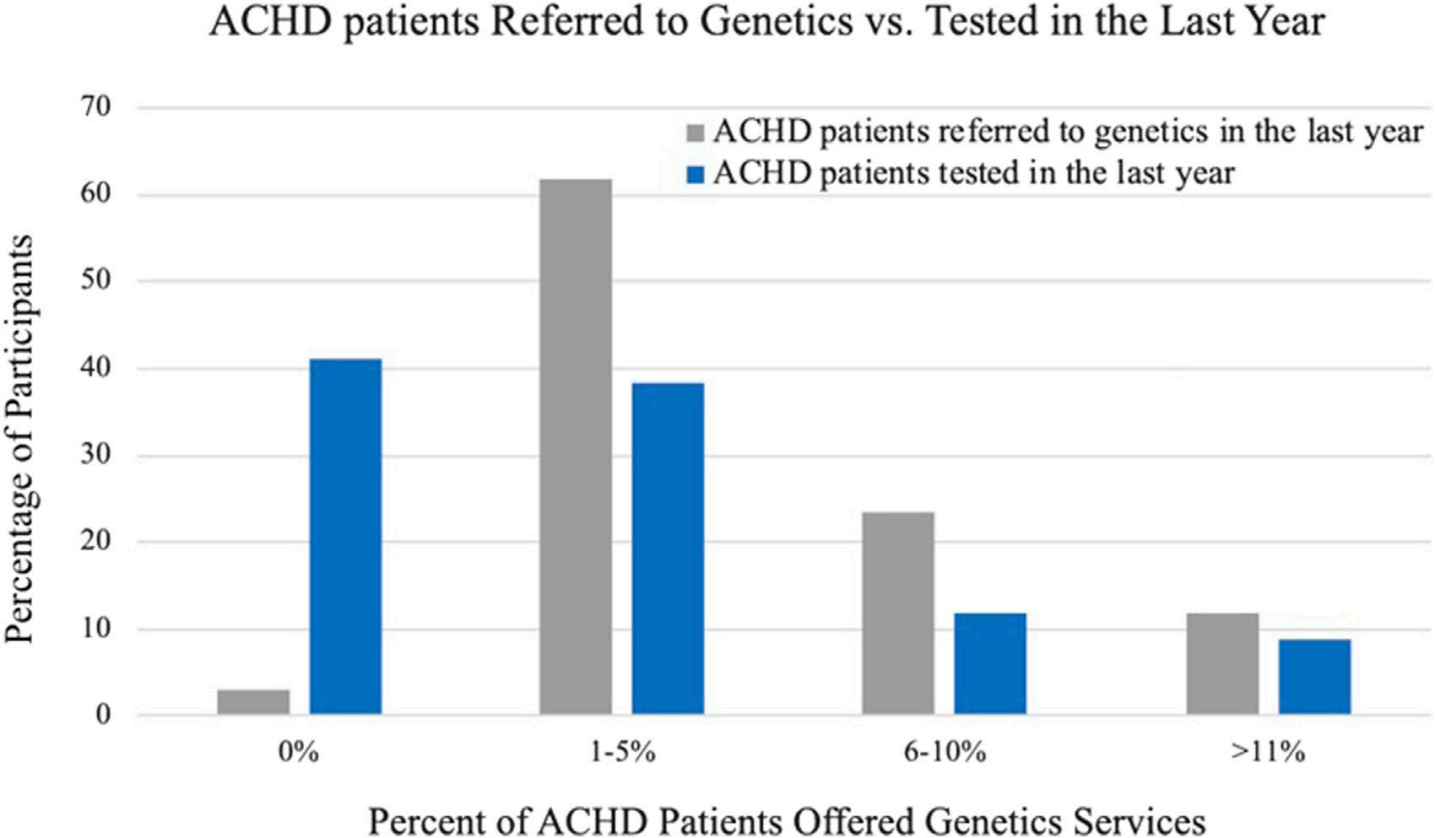
Referring and testing practices in the last year as reported by cardiologists.

The practices of cardiologists when referring and providing genetic testing in the last year are summarized in Table 3. In general, cardiologists were more likely to refer patients to a genetics professional than to order testing themselves. Nearly all cardiologists (91%) indicated that they referred one or more patients to a genetics professional in the last year and twenty cardiologists (57%) endorsed having ordered genetic testing in the last year. Of the cardiologists that did not order a genetic test in the last year, most (79%) indicated that they could easily refer to a genetics provider. Figure 1 compares the frequency of referring and ordering practices during the last year, as reported by cardiologists.

**TABLE 3:** Referral and testing practices.

	N	%
Referred one or more patients in the last year	(n = 35)	
Yes	33	94
No	1	3
No response	1	3
Ordered testing in the last year	(n = 35)	
Yes	20	57
No	14	40
No response	1	3
Type of testing ordered in the last year	(n = 20)	
Chromosome testing only	11	55
Gene sequencing only	2	10
Both chromosome testing and gene sequencing	3	15
I do not know the type of testing ordered	4	20
Type of chromosome testing ordered—*can select more than one*	(n = 14)	
FISH	13	93
Microarray	6	43
Karyotype	4	29
Unknown	1	7
Type of gene sequencing ordered—*can select more than one*	(n = 5)	
Single gene	1	20
Gene panel	3	60
Whole exome	2	40

When ordering genetic testing, most cardiologists ordered only chromosomal testing (55%) and within that subset FISH was ordered most often (93%). Of the cardiologists that ordered gene sequencing (5 cardiologists, 14%), most (60%) ordered a gene panel.

### 3.3 Risk assessment

Cardiologists were asked if they would refer four potential testing candidates to a genetics professional (Table 4). Of the four cases, the greatest number of cardiologists would refer a 30 year-old male with interrupted aortic arch, type B (n = 26, 74%).

**TABLE 4:** Referring practices based on patient presentation.

Potential genetic test candidate^a^	Cardiologists that would refer (%) n = 35
30yo male with interrupted aortic arch, type B	74
16yo male with a VSD and developmental delay	66
21yo female with heterotaxy and complex CHD	43
41yo female with transposition and family history of an ASD	40

ASD, Atrial Septal Defect VSD, Ventricular Septal Defect.

^a^ Genetics professionals would recommend that all these patients be evaluated for a genetic cause.

Cardiologists were also asked to indicate how likely they are to refer or offer testing for specific indications (Table 5). Over 90% of cardiologists indicated that they would refer if they were suspicious of a genetic syndrome (97%), if the patient requested a referral to genetics (97%), and if the patient had a finding of NDD in addition to their CHD (93%). This was very similar to the response for the indications that would be offered a genetic test. Regarding isolated/simple CHD, only 4% of cardiologists would refer to a genetics provider and only 6% would be likely to offer genetic testing.

**TABLE 5:** Likelihood of cardiologists referring or offering genetic testing.

Potential indications	Refer to genetics		Offer genetic testing	
	Mean rating^a^	% Of cardiologists likely to refer^b^	Mean rating^a^	% Of cardiologists likely to offer^b^
Suspicion of a syndrome	90	97	92	95%
Patient request	89	97	N/A	N/A
Presence of NDD	77	93	73	94%
Family history of CHD	72	84	69	71%
Extracardiac Anomaly	67	73	69	89%
Complex CHD	51	46	55	50%
Isolated/simple CHD	10	4	14	6%

NDD, Neurodevelopmental Disorders CHD, Congenital Heart Disease.

^a^ 0 = Very unlikely, 50 = Neutral, 100 = Very likely.

^b^ Respondents answering 51–100.

### 3.4 Confidence with recognizing indications for and ordering genetic testing

**FIGURE 2:**
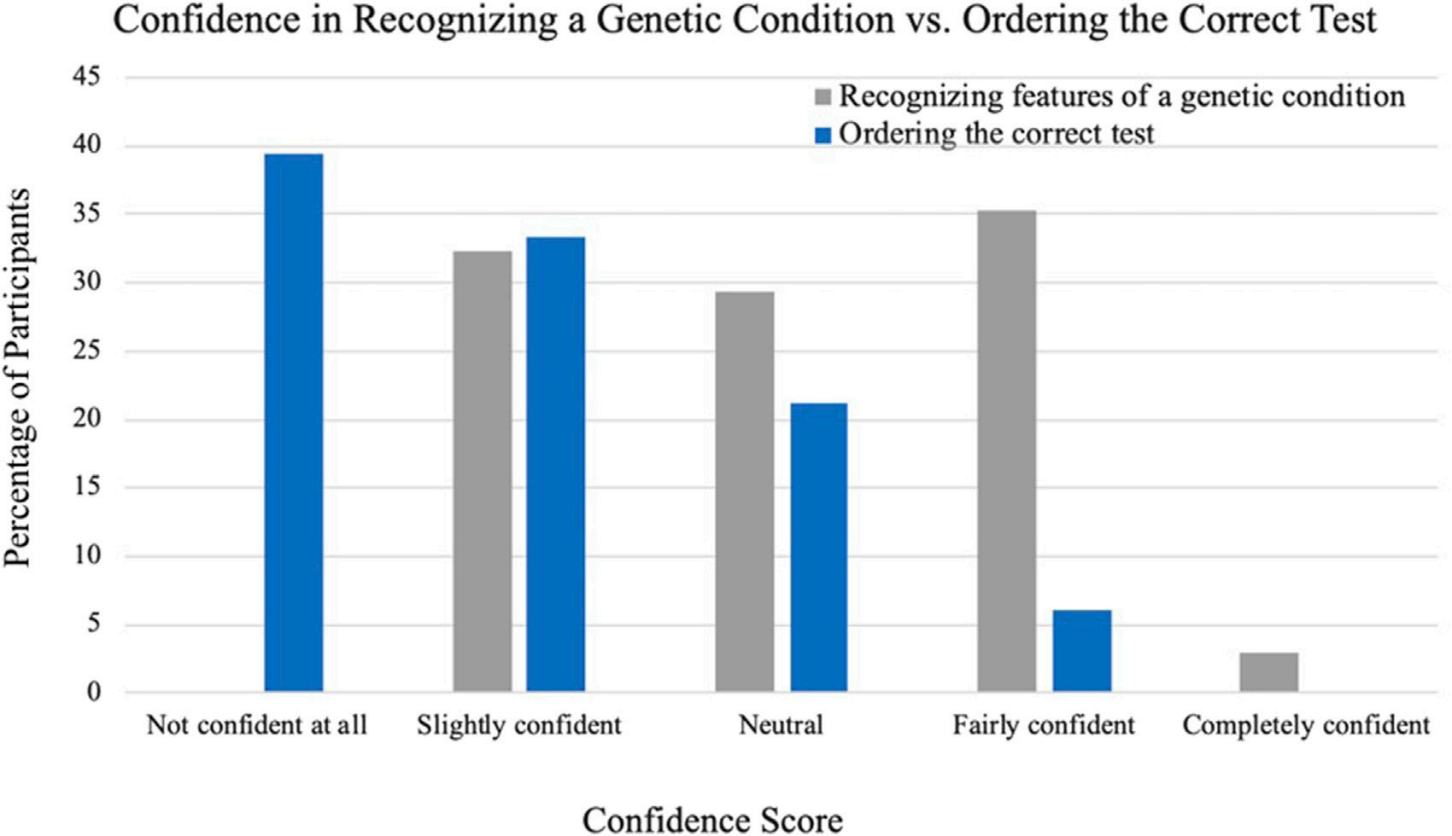
Cardiologist-reported confidence in recognizing a genetic condition compared to ordering the correct genetic test.

Cardiologists were asked their confidence level in recognizing features of a genetic condition based on patient presentations and they were then asked how confident they felt ordering the correct genetic test for their patients. Figure 2 depicts a comparison of the reported confidences between recognizing features of a genetic condition and ordering the correct genetic test. The greatest number of cardiologists endorsed being fairly confident that they could recognize a genetic condition based on patient presentation (n = 12, 35%) and no cardiologists indicated that they were not confident at all. Regarding ordering the correct test, no cardiologists endorsed being completely confident and able to assist others with ordering the correct test for ACHD and the greatest number of cardiologists indicated that they were not confident at all and would need someone else to tell them what test to order (n = 13, 39%).

### 3.5 Influential factors for placing referrals and ordering testing

The associations between cardiologist demographics, experience, barriers, genetics knowledge/confidence, and the practices reported by cardiologists are summarized in Table 6. Only knowledge scores had a significant association with referral and testing practices but there were trends in how other factors affected ordering and testing.

**TABLE 6:** Association between clinical factors and testing and referral practices.

Influential factor	Percentage of patients referred			Percentage of patients tested		
	>5%	1%–5%	*p*-value	>5%	1%–5%	*p*-value
	N (%)	N (%)		N (%)	N (%)	
Years since training			0.51			0.45
1–5 years	5 (56%)	4 (44%)		3 (50%)	3 (50%)	
6–10 years	4 (40%)	6 (60%)		3 (43%)	4 (57%)	
11–15 years	2 (25%)	6 (75%)		0 (0%)	4 (100%)	
>16 years	1 (20%)	4 (80%)		1 (33%)	2 (67%)	
Current workplace			0.67			0.33
In the United States	9 (41%)	13 (59%)		6 (46%)	7 (54%)	
Outside the United States	2 (25%)	6 (75%)		1 (17%)	5 (83%)	
Number of ACHD appointments			0.43			0.11
1–400	2 (22%)	7 (78%)		0 (0%)	5 (100%)	
>400	10 (42%)	14 (58%)		7 (47%)	8 (53%)	
Also care for pediatric CHD			0.72			1
Yes	6 (43%)	8 (57%)		3 (38%)	5 (63%)	
No	6 (32%)	13 (68%)		4 (33%)	8 (67%)	
Genetics provider in clinic			0.69			0.6
Yes	4 (44%)	5 (56%)		3 (50%)	3 (50%)	
No	7 (33%)	14 (67%)		3 (27%)	8 (73%)	
Barriers to accessing genetics			1			0.16
Yes	2 (40%)	3 (60%)		1 (13%)	7 (88%)	
No	10 (37%)	17 (63%)		6 (50%)	6 (50%)	
	Median (IQR)	Median (IQR)	*p*-value	Median (IQR)	Median (IQR)	*p*-value
Knowledge Score	1.5 (1.3–1.6)	2.0 (2.0–3.0)	0.002	1.3 (0.9–1.5)	2.0 (2.0–2.25)	0.009
Confidence recognizing features	3.0 (2.0–4.0)	3.0 (2.0–4.0)	0.83	4.0 (3.0–4.0)	3.0 (2.0–4.0)	0.12
Confidence ordering correct test	2.0 (1.75–3.0)	2.0 (1.0–2.0)	0.25	3.0 (2.5–3.0)	2.0 (1.0–2.0)	0.09

Regarding years since training, cardiologists who have been out of training longer trended toward referring less patients to genetics providers (*p* = 0.51). Of the senior ACHD cardiologists (those with more than 16 years since training) that participated, 80% reported that they refer less than 5% of the patients they see in a year. Comparatively, less than half (44%) of junior ACHD cardiologists (those with 1–5 years since training) refer 1%–5% of their patients, the majority refer more than 5% of their patients to genetics services. There also seems to be a potential association between practicing outside of the United States and testing a smaller proportion of patients (17% vs. 46%, *p* = 0.33).

Cardiologists who ordered testing on more than 5% of their patients indicated that they see more than 400 patients for ACHD per year. There also appears to be a trend toward referring more patients when cardiologists see more ACHD patients (42% vs. 22%, *p* = 0.43). Having reported barriers to offering genetic testing is associated with ordering less genetic tests (13% vs. 50%, *p* = 0.16). Evaluating pediatric patients and having a genetics professional embedded in clinic did not have an effect on referral and testing practices.

Higher scores on knowledge questions were associated with referring a *smaller* proportion of patients to genetics providers (2.0 vs. 1.5, *p* = 0.002) and ordering *less* testing (2.0 vs. 1.3, *p* = 0.009). In comparison, greater confidence in recognizing features of a genetic condition and in ordering the correct genetic test led to cardiologists ordering more genetic testing (4.0 vs. 3.0, *p* = 0.12; 3.0 vs. 2.0, *p* = 0.09).

## 4 Discussion

Understanding how cardiologists decide when to provide ACHD patients with genetic knowledge can inform management guideline development for evaluating, testing, and counseling adults with CHD. In this study we investigated cardiologists’ perspectives and practices for referring patients to genetics providers and ordering genetic testing themselves. We also explored factors that influence their decision-making.

The opportunity for cardiologists to specialize in ACHD as a subspecialty is relatively new; the American Board of Medical Specialties recognized ACHD as a separate subspecialty of cardiology in 2011 (Madan and Kim, 2015). According to the Accreditation Council for Graduate Medical Education (ACGME) website, there are currently 27 programs in the United States that offer a fellowship in ACHD. Given the small number of cardiologists specializing in ACHD, recruiting a large sample size of cardiologists for the study was difficult.

### 4.1 Utilization of genetics professionals and testing services

#### 4.1.1 Clinic structure

##### 4.1.1.1 Access to genetics services

ACHD cardiologists have a unique opportunity to connect patients with genetics services and to help them understand the possible causes of their CHD. Identifying an underlying genetic etiology can affect management, guide recurrence risk discussions, and empower patients to make informed decisions about their CHD health. A third of cardiologists in the study indicated that a genetics provider was embedded in their clinic. However, providers reported logistical challenges when placing a referral or ordering a genetic test and long wait times for genetics appointments once a referral was made.

This is indicative of a need for improved access to genetics providers evaluating ACHD patients. Potential interventions to improve access to genetics services include using electronic medical record (EMR) systems to generate referral orders and facilitate the referral process, increasing telehealth and telephone genetics appointments, and pre-visit education to increase the efficiency of genetics appointments (Bednar et al., 2022). Implementing guidelines for genetic testing in adults with CHD that are readily available to ACHD cardiologists could also streamline the process of ordering testing and referring to genetics services. In this scenario, the best test would be ordered by the cardiologist and then counseling and any additional non-cardiac management could be coordinated by the genetics providers at a follow-up visit.

##### 4.1.1.2 Affordability of genetic testing

Multiple cardiologists reported concerns about the cost of genetic testing. While affording genetic testing continues to be a challenge, the cost of testing has decreased over the last decade and resources for patients with financial need has increased (Young and Argáez, 2019). Many genetic testing laboratories also offer family variant testing to relatives at no cost, or for a lower cost than broad testing. It is possible that with more defined guidelines and criteria for genetic testing in the ACHD population, coverage of genetic testing would improve.

When ordering testing, cardiologists should discuss opportunities for financial support with their patients and assist patients in making an informed decision. If a patient endorses that cost of testing is prohibitive, it is recommended that the patient be referred to genetics to determine additional options available for the specific patient. Expertise regarding billing practices for genetic testing is one of many advantages to having geneticists or geneticist counselors involved in the provision of genetic services.

##### 4.1.1.3 Utility of a genetic diagnosis

Many cardiologists also indicated that genetic testing would not change the clinical management of their patients. One cardiologist specifically identifying post-menopausal women as a group for whom they would not offer testing, presumably because the genetic information would not inform recurrence risk in their children. Genetic evaluation offers more to patients than just management changes or recurrence risk. Limiting decision-making to direct clinical utility, overlooks the value of providing knowledge, counseling, and family testing to patients.

#### 4.1.2 Reported practices

Nearly all cardiologists reported they had referred at least one patient for genetic services in the last year but only about half ordered a genetic test. The type of testing that was ordered by cardiologists was most frequently chromosomal testing for aneuploidies and microdeletions; only five cardiologists ordered gene sequencing. Single nucleotide variants (SNVs), detected only by gene sequencing, account for about 10% of all CHD (De Backer et al., 2020). This is especially important for adults with CHD, as isolated cardiovascular defects are frequently caused by SNVs (Parrott and Ware, 2012). We would expect that for adults with syndromic CHD, the presence of extracardiac findings would have already developed and led to a genetic evaluation. Therefore, testing ACHD patients for SNVs could be more important than ordering chromosomal testing. Based on the small number of cardiologists that endorsed ordering gene sequencing, single-gene disorders associated with CHD are being under-evaluated and under-diagnosed.

The reported practices are indicative of a need for a more standardized approach to providing genetics care for adults with CHD. Providing ACHD patients with genetics services can influence patient management and decision-making, as supported by a recent study analyzing the perception of adults with CHD regarding genetic testing, preconception counseling, and family risk assessments. Participants in the study disclosed that they purposefully postponed having children until after they met with a genetics provider with some changing their plans about having a child after their genetic consultation. Most attended their genetics appointments to learn about the recurrence risk in their children and evaluate the cause of their CHD (van Engelen et al., 2013). Another important finding of the study was that most participants were referred by their cardiologist, further supporting that ACHD cardiologists have a unique opportunity to connect patients with geneticists and genetic counselors. In our study, 97% of cardiologists endorsed referring ACHD patients to genetics providers and helping their patients access genetics services.

Our understanding of the genetics of CHD is continuously evolving. For example, The Pediatric Cardiac Genomics Consortium (PCGC) researches the genetic etiologies of CHD. A recent publication from PCGC systematically evaluated CHD candidate genes and created a list of genes likely to be associated with CHD. The goal of the study was to improve the utility and yield of clinical genetic testing. Of the initial 558 candidate genes, a total of 99 genes were classified as having strong or definitive clinical validity for CHD. Furthermore, 18 of the 99 genes were associated with isolated CHD and 81 with syndromic CHD. Once genes were classified as having strong clinical validity, test results were disclosed to participants and followed up with a survey. Individuals who participated in the survey reported that understanding the cause of their CHD was important for life planning, managing future pregnancies, and improving knowledge about their diagnoses. One participant stated that having a *de novo* genetic variant was “settling” for her parents who feared they had caused her CHD (Griffin et al., 2023). The PCGC article further supports the importance of determining the underlying genetic cause of CHD for patients and their families. The reclassification of candidate CHD genes to “clinically valid” will also affect gene sequencing testing options and interpretation of results. Updated knowledge on the genetic etiologies of CHD may also warrant re-evaluation for additional genetic testing or re-interpretation of previous results such as periodic exome reanalysis.

### 4.2 Risk assessment

A genetics evaluation should be considered for any child or adult with a congenital anomaly, including CHD, given the benefits of a genetics evaluation. Additionally, many genetic conditions are characterized by incomplete penetrance and variable expressivity, making them more difficult to detect just by family history alone. Certain findings such as family history, additional congenital anomalies, and certain types of CHD may increase the yield of genetic testing. In this study, cardiologists were provided four clinical cases and asked if they would refer them to a genetics provider. All four clinical cases would benefit from a genetics evaluation, so our hypothesis was that the patients would all be referred with similar frequencies close to 100%. The results of the survey indicated that this was not the case. According to our survey, the hypothetical patient with an interrupted aortic arch would be referred by the greatest number of cardiologists. The three other cases would be referred in decreasing frequency for developmental delay, heterotaxy and complex CHD, and transposition with a positive family history. This response may be indicative of limited training on the genetic etiologies of CHD and the emphasis that is placed on referring and testing for conotruncal lesions and 22q deletion syndrome (Valente and Landzberg, 2018). The emphasis on 22q deletion syndrome may also explain why most cardiologists (n = 13/20) ordered FISH testing. Regarding ACHD training on genetic etiologies, the ACGME has two core competencies related to knowledge about the genetics of CHD and 2% of the ACHD board certification exam covers “genetic syndromes and associations.” These competencies and questions do not include specific information about diagnosing and counseling patients on the genetics of their CHD (acgme.org, abim.org).

Cardiologists also indicated that they were likely to refer and offer testing for CHD with findings other than simple/isolated CHD. Given the low rates of testing reported by cardiologists, possible reasons may include that cardiologists are 1) mostly managing patients with simple CHD or 2) referring to genetics providers when additional findings are observed, instead of ordering testing themselves. Referring patients with complex CHD or extracardiac findings to genetics professionals can provide additional education and counseling opportunities; however, if there are barriers to accessing these services, patients may not receive the recommended or requested care and go undiagnosed.

### 4.3 Confidence with genetics

Cardiologists who participated in the survey reported being more confident in recognizing features of a genetic condition than in ordering a correct genetic test. The reported referral and ordering practices reflect the difference in confidence and may explain why almost all cardiologists referred at least one patient but a much smaller subset ordered testing for patients. Most cardiologists selected that they were not confident at all and would require guidance for ordering genetic testing. Based on this result, cardiologists who do not have a genetics professional in their clinic could potentially benefit from consulting with a genetics provider to seek instruction on what testing is most appropriate, when referring to genetics is not feasible. The low confidence scores may also be indicative of a gap in ACHD training and lack of published guidance for genetic testing in adults with CHD. Therefore, we recommend an algorithm for testing in ACHD patients be developed that mirrors testing strategies used in pediatric CHD patients. Fellowship training for ACHD cardiologists and continuing medical education (CME) should also include additional education on the process of ordering testing and diagnosing genetic conditions in patients with ACHD.

### 4.4 Influential factors when deciding to refer or test

The power of our study was limited by the number of ACHD cardiologists we could invite to participate in our study, as well as our final sample size. Although we could not draw definitive conclusions, we were able to observe some trends in the associations between referral and testing practices and clinical factors. One trend we observed was that physicians who have practiced longer referred less patients and ordered less testing. Given how young the specialty of ACHD is and how quickly the field of genetics has grown, it can be expected that cardiologists who trained before the development of ACHD as a subspecialty are less likely to offer genetics services to their patients. Implementing additional education and training for ACHD cardiologists through continuing medical education (CME) could benefit physicians who trained prior to current genetic testing methodologies. It is also possible that junior cardiologist see more new patients than senior cardiologists who may follow more established patients with prior genetic testing that would not need additional testing or genetics services.

Cardiologists outside the United States indicated ordering less testing and having more barriers to testing. We investigated the availability of genetic testing outside the United States and found that as of February 2023, GeneTests^1^ listed 284 genetic testing labs in the United States and 228 non-US laboratories, mostly in Canada and Europe. There are nearly as many labs in the United States as outside the United States so the availability of testing outside the United States may be limited and dependent on the country. Therefore, the difference in testing practices indicated by cardiologists practicing outside the United States could be influenced by the availability of genetic testing labs and the types of testing offered. Testing practices outside of the United States may also be dependent on the culture of the region and the resources available to patients within a hospital system itself.

We observed that a higher number of ACHD appointments per year were associated with higher rates of genetics referrals and testing; but evaluating pediatric patients in addition to adult patients did not influence referral or testing practices. It is possible that cardiology clinics with high volumes of patients are associated with hospitals that also have high patient volumes, or with academic medical centers. In these situations, there may be more genetics providers available to consult and discuss testing. We initially anticipated that managing pediatric patients in addition to adult CHD patients would lead to higher testing and referral practices since cardiologists who cared for both populations would be familiar with pediatric CHD guidelines for making genetic referrals and providing genetic testing.

Interestingly, cardiologists who scored higher on the case-based knowledge questions in the questionnaire indicated that they referred a smaller proportion of patients and ordered less testing than their counterparts who scored lower on those questions. This association should be evaluated with caution given the small sample size of the study but it could mean that cardiologists who understand the type of testing that is indicated for specific cases are not referring or ordering as much as those who do not. It is possible that these cardiologists have resources in place to help with ordering testing and therefore they order less testing independently.

### 4.5 Conclusion

Adults with CHD are a growing population who have not had the same access to genetic services during childhood as is currently offered to children with CHD. Current genetic testing technologies and recommendations did not exist when they were children. Therefore, adults with CHD should be provided the same comprehensive access to genetic evaluation and counseling that is currently offered to infants and children. There is a need for additional guidance to increase genetics assessments in adults with CHD and other indicators of genetic risk (e.g., a non-cardiac congenital diagnosis, NDD, or family history of CHD).

The genetic etiologies of conotruncal defects are emphasized in fellowship training of cardiologists and this study supports that ACHD providers are more likely to refer and offer testing to patients with conotruncal anomalies, especially to evaluate for 22q11.2 microdeletion syndrome. For adults with CHD, testing for SNVs via gene sequencing may be of greater importance given that isolated cardiac defects are more likely to be caused by a single-gene variant and less likely to have been evaluated during childhood. Interestingly, cardiologists in this study reported ordering less gene-sequencing than chromosomal testing for their patients. Broader genetic testing for single-gene variants can also be beneficial in conditions such as Noonan Syndrome or RASopathies, where more than one gene is associated with overlapping clinical features. In these cases, adults may have a clinical diagnosis of a condition but determining what gene is affected could guide targeted testing for other family members. There is a need for improved access to genetics services and implementation of interventions such as using electronic medical record (EMR) systems to facilitate the referral process. Additional pre-visit education could increase efficiency of genetics appointments and help improve access for adults with CHD.

### 4.6 Limitations

Limitations of this study include a small sample size overall (n = 35) and a small sample of cardiologists who ordered testing in the last year (n = 20). Additionally, a response rate of 34% raises concern for potential selection bias and the generalizability of the trends identified in the sample. In the study, all the cardiologists were medical doctors (M.D.s), and most were white men working at academic medical centers. For this reason, our study may not be generalizable to all cardiologists who evaluate patients for ACHD. There is limited information on the demographics of ACHD cardiologists, but according to a Professional Life Survey (PLS) of U.S. cardiologists conducted in 2015 by the American College of Cardiology (ACC), 64% of cardiologists were white and 58% were men (Thomas et al., 2021). By surveying cardiologists outside of the United States we tried to increase the diversity of the study and capture differences in referral and testing practices but our questionnaire did not include questions specific to cardiologists practicing outside of the United States.

### 4.7 Future research

A similar provider survey could be used to evaluate how pediatric cardiologists offer genetics services to children with CHD, identify the indications that influence decision-making for genetics care, and determine if published recommendations for genetics evaluations in children with CHD are being utilized by providers. Further investigation on how ACHD fellows are trained on the genetic etiologies of CHD, especially single-gene disorders, and possible educational tools that could fill in the knowledge gap is warranted based on the findings of this study. A study focused on ACHD provider knowledge and confidence recognizing a genetic syndrome and ordering testing would provide a more robust evaluation than what we were able to achieve in this study.

## Data Availability

The original contributions presented in the study are included in the article/supplementary material, further inquiries can be directed to the corresponding author.

## Footnotes

1 GeneTests is federally funded and includes an international directory of laboratories offering clinical and research genetic testing; it is maintained by the University of Washington, Seattle, Washington

## References

[B1] Bednar E. M. Nitecki R. Krause K. J. Rauh-Hain J. A. (2022). Interventions to improve delivery of cancer genetics services in the United States: a scoping review. official J. Am. Coll. Med. Genet. 24 (6), 1176–1186. 10.1016/j.gim.2022.03.002 PMC12234025 35389342

[B2] Bernier F. P. Spaetgens R. (2006). The geneticist's role in adult congenital heart disease. Cardiol. Clin. 24 (4), 557–569. 10.1016/j.ccl.2006.08.001 17098511

[B3] Botto L. D. Lin A. E. Riehle-Colarusso T. Malik S. Correa A. National Birth Defects Prevention Study (2007). Seeking causes: classifying and evaluating congenital heart defects in etiologic studies. Clin. Mol. Teratol. 79 (10), 714–727. 10.1002/bdra.20403 17729292

[B4] Bouma B. J. Mulder B. J. (2017). Changing landscape of congenital heart disease. Circulation Res. 120 (6), 908–922. 10.1161/CIRCRESAHA.116.309302 28302739

[B5] Boynton P. M. Greenhalgh T. (2004). Selecting, designing, and developing your questionnaire. BMJ Clin. Res. ed. 328 (7451), 1312–1315. 10.1136/bmj.328.7451.1312 PMC420179 15166072

[B6] Bracher I. Padrutt M. Bonassin F. Santos Lopes B. Gruner C. Stämpfli S. F. (2017). Burden and impact of congenital syndromes and comorbidities among adults with congenital heart disease. Int. J. Cardiol. 240, 159–164. 10.1016/j.ijcard.2017.02.118 28606676

[B7] Chaix M. A. Andelfinger G. Khairy P. (2016). Genetic testing in congenital heart disease: a clinical approach. World J. Cardiol. 8 (2), 180–191. 10.4330/wjc.v8.i2.180 26981213 PMC4766268

[B8] Culver J. O. Hull J. L. Dunne D. F. Burke W. (2001). Oncologists' opinions on genetic testing for breast and ovarian cancer. official J. Am. Coll. Med. Genet. 3 (2), 120–125. 10.1097/00125817-200103000-00006 11280949

[B9] De Backer J. Bondue A. Budts W. Evangelista A. Gallego P. Jondeau G. (2020). Genetic counselling and testing in adults with congenital heart disease: a consensus document of the esc working group of grown-up congenital heart disease, the esc working group on aorta and peripheral vascular disease and the European society of human genetics. Eur. J. Prev. Cardiol. 27 (13), 1423–1435. 10.1177/2047487319854552 31184212

[B10] Gaeta S. A. Ward C. Krasuski R. A. (2016). Extra-cardiac manifestations of adult congenital heart disease. Trends Cardiovasc. Med. 26 (7), 627–636. 10.1016/j.tcm.2016.04.004 27234354

[B11] Geddes G. C. Earing M. G. (2018). Genetic evaluation of patients with congenital heart disease. Curr. Opin. Pediatr. 30 (6), 707–713. 10.1097/MOP.0000000000000682 30138133 PMC6257101

[B12] Griffin E. L. Nees S. N. Morton S. U. Wynn J. Patel N. Jobanputra V. (2023). Evidence-based assessment of congenital heart disease genes to enable returning results in a genomic study. Genomic Precis. Med. 16, e003791. 10.1161/CIRCGEN.122.003791 PMC10121846 36803080

[B13] Harris P. A. Taylor R. Minor B. L. Elliott V. Fernandez M. O'Neal L. (2019). The REDCap consortium: building an international community of software platform partners. J. Biomed. Inf. 95, 103208. 10.1016/j.jbi.2019.103208 PMC7254481 31078660

[B14] Harris P. A. Taylor R. Thielke R. Payne J. Gonzalez N. Conde J. G. (2009). Research electronic data capture (REDCap)--a metadata-driven methodology and workflow process for providing translational research informatics support. J. Biomed. Inf. 42 (2), 377–381. 10.1016/j.jbi.2008.08.010 PMC2700030 18929686

[B15] Ison H. E. Griffin E. L. Parrott A. Shikany A. R. Meyers L. Thomas M. J. (2021). Genetic counseling for congenital heart disease - practice resource of the national society of genetic counselors. J. Genet. Couns. 31, 9–33. 10.1002/jgc4.1498 34510635

[B16] Ito S. Chapman K. A. Kisling M. John A. S. (2017). Appropriate use of genetic testing in congenital heart disease patients. Curr. Cardiol. Rep. 19 (3), 24. 10.1007/s11886-017-0834-1 28224467

[B17] Kiess M. (2016). History and evolution of the treatment of adult congenital heart disease. BCMJ 58 (7), 368–372.

[B18] Landstrom A. P. Kim J. J. Gelb B. D. Helm B. M. Kannankeril P. J. Semsarian C. (2021). Genetic testing for heritable cardiovascular diseases in pediatric patients: a scientific statement from the American heart association. Circ. Genom Precis. Med. 14 (5), e000086. 10.1161/HCG.0000000000000086 34412507 PMC8546375

[B19] Lapum J. L. Fredericks S. Bailey B. Yau T. M. Graham J. Marelli A. J. (2019). Historical investigation of medical treatment for adult congenital heart disease: a Canadian perspective. Congenit. heart Dis. 14 (2), 185–192. 10.1111/chd.12716 30451387

[B20] Madan P. Kim Y. Y. (2015). Training in adult congenital heart disease. J. Am. Coll. Cardiol. 65 (20), 2254–2256. 10.1016/j.jacc.2015.04.002 25998671

[B21] Mazor Dray E. Marelli A. J. (2015). Adult congenital heart disease: scope of the problem. Cardiol. Clin. 33 (4), 503–512. 10.1016/j.ccl.2015.07.001 26471815

[B22] Miller D. T. Adam M. P. Aradhya S. Biesecker L. G. Brothman A. R. Carter N. P. (2010). Consensus statement: chromosomal microarray is a first-tier clinical diagnostic test for individuals with developmental disabilities or congenital anomalies. Am. J. Hum. Genet. 86, 749–764. 10.1016/j.ajhg.2010.04.006 20466091 PMC2869000

[B23] Nees S. N. Chung W. K. (2020). The genetics of isolated congenital heart disease. Am. J. Med. Genet. Part C, Seminars Med. Genet. 184 (1), 97–106. 10.1002/ajmg.c.31763 PMC8211463 31876989

[B24] Parrott A. Ware S. M. (2012). The role of the geneticist and genetic counselor in an ACHD clinic. Prog. Pediatr. Cardiol. 34 (1), 15–20. 10.1016/j.ppedcard.2012.05.004 23049235 PMC3462440

[B25] Pierpont M. E. Basson C. T. Benson D. W. Jr Gelb B. D. Giglia T. M. Goldmuntz E. (2007). Genetic basis for congenital heart defects: current knowledge: a scientific statement from the American heart association congenital cardiac defects committee, Council on cardiovascular disease in the young: endorsed by the American academy of pediatrics. Circulation 115 (23), 3015–3038. 10.1161/CIRCULATIONAHA.106.183056 17519398

[B26] Pierpont M. E. Brueckner M. Chung W. K. Garg V. Lacro R. V. McGuire A. L. (2018). Genetic basis for congenital heart disease: revisited: a scientific statement from the American heart association. Circulation 138, e653–e711. 10.1161/CIR.0000000000000606 30571578 PMC6555769

[B27] Prochniak C. F. Martin L. J. Miller E. M. Knapke S. C. (2012). Barriers to and motivations for physician referral of patients to cancer genetics clinics. J. Genet. Couns. 21 (2), 305–325. 10.1007/s10897-011-9401-x 21842318

[B28] Rimoin D. Hirschhorn K. A. (2004). A history of medical genetics in pediatrics. Pediatr. Res. 56, 150–159. 10.1203/01.PDR.0000129659.32875.84 15128921

[B29] Shikany A. R. Landis B. J. Parrott A. Miller E. M. Coyan A. Walters L. (2020). A comprehensive clinical genetics approach to critical congenital heart disease in infancy. J. Pediatr. 227, 231–238. 10.1016/j.jpeds.2020.07.065 32717230 PMC8424561

[B30] Stout K. K. Daniels C. J. Aboulhosn J. A. Bozkurt B. Broberg C. S. Colman J. M. (2019). 2018 AHA/ACC guideline for the management of adults with congenital heart disease: a report of the American College of cardiology/American heart association task force on clinical practice guidelines. J. Am. Coll. Cardiol. 73 (12), e81–e192. 10.1016/j.jacc.2018.08.1029 30121239

[B31] Thomas K. L. Mehta L. S. Rzeszut A. K. Lewis S. J. Duvernoy C. S. Douglas P. S. ACC Diversity and Inclusion Task Force and ACC Women in Cardiology Section (2021). Perspectives of racially and ethnically diverse U.S. Cardiologists: insights from the ACC professional life survey. J. Am. Coll. Cardiol. 78 (17), 1746–1750. 10.1016/j.jacc.2021.09.002 34674820

[B32] Valente A. Landzberg M. J. (2018). “Congenital heart disease in the adult,” in Harrison's principles of internal medicine. Editors Jameson J. Fauci A. S. Kasper D. L. Hauser S. L. Longo D. L. Loscalzo J. (New York City, NY, USA: McGraw Hill). Available at: https://accesspharmacy.mhmedical.com/content.aspx?bookid=2129&sectionid=189405859.

[B33] van der Bom T. Zomer A. C. Zwinderman A. H. Meijboom F. J. Bouma B. J. Mulder B. J. (2011). The changing epidemiology of congenital heart disease. Nat. Rev. Cardiol. 8 (1), 50–60. 10.1038/nrcardio.2010.166 21045784

[B34] van Engelen K. Baars M. J. Felix J. P. Postma A. V. Mulder B. J. Smets E. M. (2013). The value of the clinical geneticist caring for adults with congenital heart disease: diagnostic yield and patients' perspective. Am. J. Med. Genet. A 161A (7), 1628–1637. 10.1002/ajmg.a.35973 23696448

[B35] van Engelen K. Baars M. J. van Rongen L. T. van der Velde E. T. Mulder B. J. Smets E. M. (2011). Adults with congenital heart disease: patients' knowledge and concerns about inheritance. Am. J. Med. Genet. A 155A (7), 1661–1667. 10.1002/ajmg.a.34068 21671389

[B36] Young C. Argáez C. (2019) “Rapid genome-wide testing: a review of clinical utility, cost-effectiveness, and guidelines,” in Canadian agency for drugs and technologies in health. 31721549

